# Impact of the route of adrenaline administration in patients suffering from out-of-hospital cardiac arrest on 30-day survival with good neurological outcome (ETIVIO study)

**DOI:** 10.1186/s13049-023-01079-9

**Published:** 2023-03-30

**Authors:** Tobias Monaco, Matthias Fischer, Mark Michael, Iryna Hubar, Ralf Westenfeld, Stefan Rauch, Jan-Thorsten Gräsner, Michael Bernhard

**Affiliations:** 1 Emergency Department, University Hospital of Düsseldorf, Heinrich Heine University, Moorenstrasse 5, D-40225 Düsseldorf, Germany; 2 Department of Anaesthesiology and Intensive Care, ALB FILS Kliniken, Eichertstraße 3, 73035 Göppingen, Germany; 3 Division of Cardiology, Pulmonology and Vascular Medicine, Medical Faculty, University Hsopital of Düsseldorf, Heinrich Heine University, Moorenstrasse 5, 40225 Düsseldorf, Germany; 4 Institute for Emergency Medicine, Department of Anesthesiology and Intensive Care Medicine, University-Hospital Schleswig-Holstein, Arnold-Heller-Straße 3, 24105 Kiel, Germany

**Keywords:** Out-of-hospital cardiac arrest, Adrenaline, Route of drug administration, Intravenous access, Intraosseous access, Endotracheal access

## Abstract

**Background:**

Over the past decades, international guidelines for cardiopulmonary resuscitation (CPR) have changed the recommendation for alternative routes for drug administration. Until now, evidence for the substantial superiority of one route with respect to treatment outcome after CPR has been lacking. The present study compares the effects of intravenous (IV), intraosseous (IO) and endotracheal (ET) adrenaline application during CPR in out-of-hospital cardiac arrest (OHCA) on clinical outcomes within the database of the German Resuscitation Registry (GRR).

**Methods:**

This registry analysis was based on the GRR cohort of 212,228 OHCA patients between 1989 and 2020. Inclusion criteria were: OHCA, application of adrenaline, and out-of-hospital CPR. Excluded from the study were patients younger than 18 years, those who had trauma or bleeding as suspected causes of cardiac arrest, and incomplete data sets. The clinical endpoint was hospital discharge with good neurological outcome [cerebral performance category (CPC) 1/2]. Four routes of adrenaline administration were compared: IV, IO, IO + IV, ET + IV. Group comparisons were done using matched-pair analysis and binary logistic regression.

**Results:**

In matched-pair group comparisons of the primary clinical outcome hospital discharge with CPC 1/2, the IV group (n = 2416) showed better results compared to IO (n = 1208), [odds ratio (OR): 2.43, 95% confidence interval (CI): 1.54–3.84, p < 0.01] and when comparing IV (n = 8706) to IO + IV (n = 4353), [OR: 1.33, 95% CI: 1.12–1.59, p < 0.01]. In contrast, no significant difference was found between IV (n = 532) and ET + IV (n = 266), [OR: 1.26, 95% CI: 0.55–2.90, p = 0.59]. Concurrently, binary logistic regression yielded a highly significant effect of vascular access type (χ² = 67.744(3), p < 0.001) on hospital discharge with CPC1/2, with negative effects for IO (regression coefficient (r.c.) = − 0.766, p = 0.001) and IO + IV (r.c. = − 0.201, p = 0,028) and no significant effect for ET + IV (r.c. = 0.117, p = 0.770) compared to IV.

**Conclusions:**

The GRR data, collected over a period of 31 years, seem to emphasize the relevance of an IV access during out-of-hospital CPR, in the event that adrenaline had to be administered. IO administration of adrenaline might be less effective. ET application, though removed in 2010 from international guidelines, could gain importance as an alternative route again.

## Introduction

Sudden out-of-hospital cardiac arrest (OHCA) is the third leading cause of death in Europe [1]. According to the results of the European Registry of Cardiac Arrest (EuReCa) ONE trial, 30-day survival is at 10% [1]. The three-month EuReCa TWO trial showed for data of 25,171 patients an OHCA incidence of 56 per 100,000 inhabitants, with a return of spontaneous circulation (ROSC) rate of 33% and a hospital discharge rate of 8% [2].

According to the international guidelines for advanced life support (ALS) by the European Resuscitation Council (ERC), the administration of adrenaline (epinephrine) is part of recommended standard actions during cardiopulmonary resuscitation (CPR) for both shockable rhythms (ventricular fibrillation and pulseless ventricular tachycardia) and non-shockable rhythms (asystole and pulseless electrical activity) in the out-of-hospital setting [3].

However, it remains unclear through which route of administration adrenaline seems to be most beneficial for overall survival and clinical outcome after OHCA. The gold standard for adrenaline application is the intravenous (IV) access [3], while the intraosseous (IO) access provides an alternative route. For Germany, a national guideline is available for IO infusion within emergency settings [4]. Therefore, in order to ensure quick drug and fluid resuscitation despite insufficient venous conditions, nearly all out-of-hospital rescue vehicles have been equipped with IO access devices. In 2010, endotracheal administration (ET) was removed from international recommendations.

IO devices have thus been established as effective tools in various emergency settings. However, due to the obvious ethical and practical limitations that come with researching CPR, evidence remains scarce as to the effects of various routes of drug administration during CPR within the particularly demanding setting of OHCA.

Therefore, this study analyzes the available registry data from the German Resuscitation Registry (GRR) to determine whether application routes are associated with effects on clinical outcomes, namely ROSC and survival with good neurological outcome. The results will allow for international comparisons with other physician-based emergency medical systems (EMS). Additionally, the analysis of a currently not recommended route – endotracheal administration – will be provided.

## Materials and methods

### German resuscitation Registry

This study was designed as a registry analysis of all OHCA compiled in the GRR between 1989 and 2020. The GRR is a prospective registry, maintained by the German Society of Anesthesiology and Intensive Care Medicine. It covers 30 million inhabitants in Germany [5] and 1.2 million inhabitants in Austria (unpublished for 2020) with comparable physician-based out-of-hospital emergency health care systems [6]. All participating EMS dispatch both paramedic-staffed ambulances and physician-staffed vehicles to suspected OHCA cases. The design of the GRR follows the Utstein style [7]. Registry participation is voluntary. Data entries are carried out by EMS physicians or other EMS staff and have to be cleared by the responsible chief medical officer. In order to maintain overall database consistency and to minimize selection bias, only data from ambulance services meeting the following criteria were added to the present analysis: yearly OHCA prevalence of at least 30 per 100,000 inhabitants, mean ROSC rate under 80%, ROSC after cardiac arrest (RACA) score availability above 60%, follow-up data documenting post-admission outcomes for at least 30% of cases. The RACA score [8] provides one method to assess the likelihood for ROSC after cardiac arrest. Cases from ambulance services not meeting the quality criteria were excluded from further analysis, especially when long-term outcome could not be assessed due to lacking follow-up data.

### Inclusion criteria

**Fig. 1:**
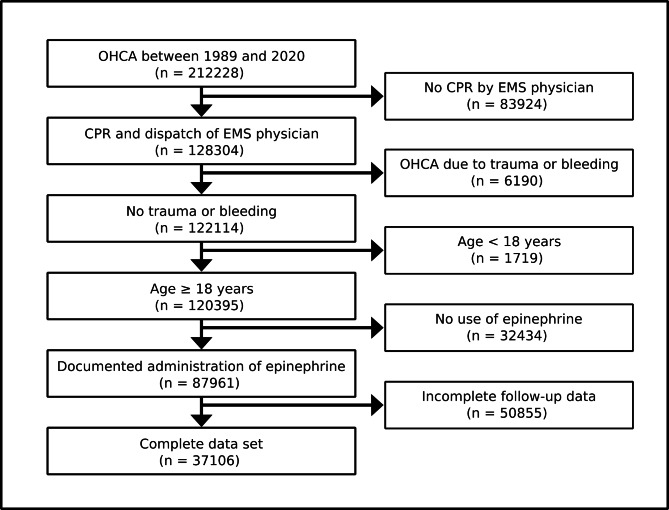
Inclusion and exclusion criteria. OHCA: out-of-hospital cardiac arrest, CPR: cardiopulmonary resuscitation, EMS: emergency medical service

The analysis was based on 212,228 anonymous data sets of adult patients with OHCA. Further inclusion criteria were CPR – independent of the initiation by bystanders or EMS personnel – and the administration of adrenaline by EMS (Fig. 1).

### Exclusion criteria

The exclusion criteria were age < 18 years, trauma or bleeding as suspected causes of cardiac arrest and incomplete data sets (Fig. 1).

### Primary and secondary endpoints

Primary endpoint: discharge with good neurological outcome, defined as cerebral performance category (CPC) 1 or 2 (Table 1). Secondary endpoints: ROSC during out-of-hospital care, survival at hospital admission or admission under ongoing CPR, 24 h survival, and survival at hospital discharge or 30 day survival.

**Table 1:** Outcome Parameters

**Primary Endpoint:**
• Discharge with good neurological outcome (CPC 1 or 2)
**Secondary Endpoints**:
• ROSC during out-of-hospital care • Survival (ROSC) at hospital admission or admission under ongoing CPR • 24 h survival • Survival at hospital discharge or 30d-survival
**Additional Parameters**:
• age (years) • gender (m/f/n) • etiology of cardiac arrest (non-traumatic vs. traumatic) • witness of cardiac arrest (no witness, lay-person, EMS personnel) • initial heart rhythm (v-fib,v-tach, asystole, PEA) • bystander-CPR (yes/no) • EMS response time (minutes) in groups • duration of resuscitation (EMS on-site arrival until hospital admission in minutes) • out-of-hospital administration of medication (e.g. adrenaline, amiodarone) with dose, frequency and route (IV, IO, ET, IO + IV, ET + IV, IO + ET + IV)

### Additional parameters

The following data were also acquired and used for inclusion, exclusion and risk-adjusted pair-matching: age (years), sex (male, female), etiology of cardiac arrest (non-traumatic, traumatic), pre-emergency status (no/minor/major/severe/unknown prior disease), initial heart rhythm (ventricular fibrillation, ventricular tachycardia, asystole, pulseless electrical activity), bystander-CPR (yes, no), EMS response time (minutes), duration of resuscitation (EMS on-site arrival until hospital admission in minutes), out-of-hospital administration of medication (e.g. adrenaline, amiodarone) with dose, frequency and route (IV, IO, ET, IO + IV, ET + IV, IO + ET + IV), deltaROSC: the difference between observed ROSC and ROSC after cardiac arrest (RACA) score [8].

### Group definitions

Patients were pooled in four groups regarding the route of adrenaline administration: IV access, IO access, IO followed by IV access (IO + IV), and ET followed by IV access (ET + IV). Outcomes were analyzed for three group contrasts after risk-adjustment through pair-matching: IV vs. IO, IV vs. IO + IV, and IV vs. ET + IV.

### Data processing and statistical analysis

Anonymous registry entries were processed in Microsoft Excel 365 MSO 16.0 64-Bit (Microsoft, Redmond, WA) and analyzed with IBM SPSS Statistics 26 (IBM, Armonk, NY), using Student’s two-sided t-test for parametric data and the χ^2^-test for non-parametric variables. Statistical significance was assumed for *p*-values below or equal 0.05.

In order to minimize confounding and selection bias, group comparisons were performed by matched-pair analysis including the following variables, known to affect clinical outcome after OHCA [8]: time from emergency call to arrival of EMS, percentage of shockable rhythms (ventricular fibrillation or ventricular tachycardia), asystole, cardiogenic cause, hypoxia, OHCA witnessed by bystander, OHCA witnessed by EMS, bystander CPR, age above 80 years, age between 18 and 65 years, OHCA in public or at doctor’s office, OHCA at home, OHCA at nursing home, sex, initial electrocardiogram.

Confounder corrected group analysis was achieved by matched-pair group comparisons via the custom-built software PairMatcher [9, 10]. Due to its larger size, the IV group was matched 2:1 with all other groups, i.e. two IV patients were matched with respect to all control variables with one patient each of the IO, IO + IV, and ET + IV group respectively. As internal validation for adequate pair-matching, the ROSC after cardiac arrest (RACA) score [8], derived from multivariate logistic regression to predict likelihood of ROSC after OHCA, was calculated for each group contrast, confirming the clinical comparability of the matched groups prior to further analysis.

A secondary regression analysis was performed to assess the amount of variance explained by vascular access type. Hospital discharge with good neurological outcome was set as clinical outcome parameter. A binary logistic regression model with vascular access type as independent variable was calculated through SPSS, taking all above-mentioned parameters of the pair-matching approach into account, and additionally correcting for age, adrenaline dosage, intervention with coronary catheter and treatment with mild therapeutic hypothermia during hospital stay.

## Results

### Descriptive statistics

During the study period between 1989 and 2020, the analysis of the GRR database revealed 212,228 cases of OHCA. After application of the aforementioned inclusion and exclusion criteria, 37,106 complete data sets were subjected to further analysis (Fig. 1). Of those OHCA patients, 29,688 had received an IV access, 1,303 an IO access, 4,827 both IO and IV accesses and 276 patients had received both ET and IV therapy (Table 2). 20 patients had received adrenaline exclusively via ET, 5 via ET and IO, and 23 via a combination of ET, IO and IV accesses. For 964 cases, no route of drug administration was documented.

**Table 2:** Descriptive statistics and matched-pair analysis with outcome depending on route of administration. IV: all included patients with intravenous access, IO: all included patients with intraosseous access, IO + IV: all patients with both IO and IV access, ET + IV: all patients with both endotracheal and IV access, ET: all patients with only ET access, ET + IO: all patients with both ET and IO access, ET + IO + IV: all patients with all three access routes, n.sp.: not specified, IV_[IO]_: IV-subgroup to match IO, IV_[IO+IV]_: IV-subgroup to match IO + IV, IV_[ET+IV]_: IV-subgroup to match ET + IV, n: number of patients included, ROSC: return of spontaneous circulation with 95% confidence interval, VF/VT: ventricular fibrillation or ventricular tachycardia, Cardiogenic: cardiogenic cause of OHCA, Hypoxia: hypoxia as cause of OHCA, Witn. Byst.: OHCA witnessed by bystander, Witn. EMS: OHCA witnessed by EMS personnel, Byst. CPR: CPR performed by bystander, In public: OHCA in public or at doctor’s office, At home: OHCA at private home, Nurs. home: OHCA in a nursing facility, Time to EMS arrival: time from alarm until arrival of first EMS vehicle in minutes:seconds with standard deviation, MTH: percentage of hospitalized patients receiving mild therapeutic hypothermia, Cardiac cath.: percentage of hospitalized patients receiving cardiac catheter intervention, RACA: ROSC after cardiac arrest score, Δ-RACA: ROSC-RACA, hence the difference between actual ROSC and mean predicted ROSC by RACA score, CPR@ED: admission to emergency department under cardiopulmonary resuscitation, ROSC@ED: admission to ED after ROSC, 24 h survival: rate of patients alive 24 h after admission, 30d survival: rate of patients alive or discharged 30 days after admission, CPC 1/2: cerebral performance category 1 or 2

Patient characteristics										Matched pairs			Matched pairs			Matched pairs		
	All cases	IV	IO	IO + IV	ET + IV	ET	ET + IO	ET + IO + IV	n.sp.	IV_[IO]_	IO	Chi²/t	IV_[IO+IV]_	IO + IV	Chi²/t	IV_[ET+IV]_	ET + IV	Chi²/t
Number (n)	37,106	29,688	1303	4827	276	20	5	23	964	2416	1208	–	8706	4353	–	532	266	–
ROSC (%) 95% CI	45.6 (45.1,46.1)	46.8 (46.2,47.4)	35.4 (32.8,38.1)	42.1 (40.7,43.5)	40.2 (34.4,46.3)	15.0 (3.2,37.9)	20.0 (0.5,71.6)	34.8 (16.4,57.3)	42.0 (38.9,45.2)	44.1 (42.1,46.1)	34.9 (32.2,37.7)	< 0.001	45.0 (44.0,46.1)	41.2 (39.7,42.7)	< 0.001	43.2 (39.0,47.6)	40.2 (34.3,46.4)	0.418
VF/VT (%)	23.9	25.3	15.1	18.5	19.6	10.0	0.0	34.8	20.5	15.1	15.1	1.0	18.8	18.8	1.0	19.9	19.9	1.0
Asystole (%)	54.0	52.7	63.1	58.2	61.6	65.0	40.0	47.8	57.2	64.4	64.4	1.0	59.1	59.1	1.0	61.3	61.3	1.0
Cardiogenic (%)	65.8	67.3	53.3	63.1	53.6	45.0	60.0	52.2	54.1	79.7	79.7	1.0	82.5	82.5	1.0	90.2	90.2	1.0
Hypoxia (%)	12.8	12.2	17.0	15.9	7.3	5.0	0.0	17.4	11.2	15.2	15.2	1.0	13.4	13.4	1.0	5.6	5.6	1.0
Witn. Byst. (%)	44.1	44.8	39.0	42.8	37.7	35.0	20.0	52.2	37.5	38.3	38.3	1.0	43.0	43.0	1.0	38.0	38.0	1.0
Witn. EMS (%)	7.2	7.6	6.0	5.6	3.6	0.0	20.0	4.4	7.0	5.1	5.1	1.0	4.7	4.7	1.0	2.6	2.6	1.0
Byst. CPR (%)	33.3	33.6	33.4	34.3	12.7	15.0	0.0	26.1	26.5	32.4	32.4	1.0	33.8	33.8	1.0	12.0	12.0	1.0
> 80 years (%)	28.2	29.1	23.3	23.9	27.5	20.0	0.0	13.0	29.9	23.5	23.5	1.0	24.7	24.7	1.0	27.4	27.4	1.0
18–65 years (%)	33.6	32.5	40.1	38.8	33.7	35.0	80.0	60.9	32.9	39.2	39.2	1.0	37.3	37.3	1.0	33.1	33.1	1.0
In public (%)	19.5	19.7	18.3	19.0	16.7	15.0	0.0	17.4	19.3	18.0	18.0	1.0	18.5	18.5	1.0	16.5	16.5	1.0
At home (%)	65.2	65.1	66.8	66.5	70.0	70.0	100	56.5	59.8	70.0	70.0	1.0	69.5	69.5	1.0	71.1	71.1	1.0
Nurs. home (%)	9.2	9.1	9.5	9.9	5.8	10.0	0.0	4.4	7.9	7.7	7.7	1.0	8.7	8.7	1.0	4.9	4.9	1.0
Male (%)	65.9	66.7	62.3	62.2	65.9	60.0	60.0	47.8	64.5	63.1	63.1	1.0	64.0	64.0	1.0	66.2	66.2	1.0
Time to EMS arrival ± st.d.	6:21 ± 3:23	06:21 ± 3:24	6:00 ± 3:13	6:21 ± 3:20	6:09 ± 3:04	5:07 ± 2:35	6:30 ± 2:07	5:42 ± 3:08	6:38 ± 3:41	6:27 ± 3:23	5:56 ± 3:08	< 0.001	6:23 ± 3:26	6:21 ± 3:18	0.480	6:08 ± 2:57	6:07 ± 3:04	0.918
MTH (%)	26.6	27.3	19.2	25.0	14.0	25.0	0.0	50.0	24.5	25.0	19.1	0.010	27.9	25.7	0.070	19.3	14.6	0.262
Cardiac cath. (%)	28.5	28.9	21.6	28.4	14.7	25.0	0.0	37.5	25.0	26.1	22.6	0.139	29.0	30.1	0.350	15.5	15.5	0.983
RACA score (%)	43.2	43.5	41.5	42.6	39.1	38.5	52.5	50.5	42.3	40.6	41.0	0.483	41.6	41.9	0.382	39.4	38.8	0.590
Δ-RACA (%)	2.3	3.3	-6.2	-0.6	1.1	-23.5	-32.5	-15.7	-0.3	3.5	-6.1	–	3.4	-0.7	–	3.8	1.4	–
CPR@ED (%)	13.2	12.9	13.9	15.6	11.6	5.0	40.0	8.7	12.7	11.6	13.6	0.092	12.0	15.1	< 0.001	12.6	11.3	0.592
ROSC@ED (%)	36.5	37.9	26.6	31.6	35.1	15.0	20.0	26.1	32.2	35.6	26.3	< 0.001	35.8	30.7	< 0.001	37.0	35.0	0.567
24 h survival (%)	21.5	22.4	16.4	18.1	17.4	5.0	40.0	21.7	18.5	20.0	16.1	0.005	21.3	17.5	< 0.001	20.1	17.7	0.410
30d survival (%)	9.2	9.8	4.8	7.2	6.2	–	–	8.7	7.9	7.6	4.7	0.001	8.9	7.0	< 0.001	7.0	6.4	0.765
CPC 1/2 (%)	5.5	6.0	1.8	4.1	2.9	–	–	8.7	5.3	4.5	1.9	< 0.001	5.3	4.0	0.002	3.8	3.0	0.586

Remarkably, all groups with sufficient data (IV, IO, IO + IV, ET + IV) showed RACA scores of comparable magnitudes, centering around a mean ± SD of 41.7% ±1.9, suggesting roughly equal pre-CPR conditions on average. The actual ROSC rates in contrast were more than twice as variable with a mean ± SD of 41.1% ±4.7.

Group effects of route of adrenaline administration on clinical outcomes were calculated after separate pairwise matching of every IO, IO + IV and ET + IV case with two IV cases each with comparable pre-CPR OHCA conditions.

### Internal validation

Table 2 shows that the average RACA score of each IV-subgroup closely matched the respective comparison group with no divergence exceeding 0.6%. This confirmed the intended matching procedure. Differences between the various IV subgroups were an expected effect of the pair-matching procedure, reflecting pre-CPR differences between the matched IO, IO + IV and ET + IV groups.

### Statistical analysis of primary and secondary endpoints

**Fig. 2:**
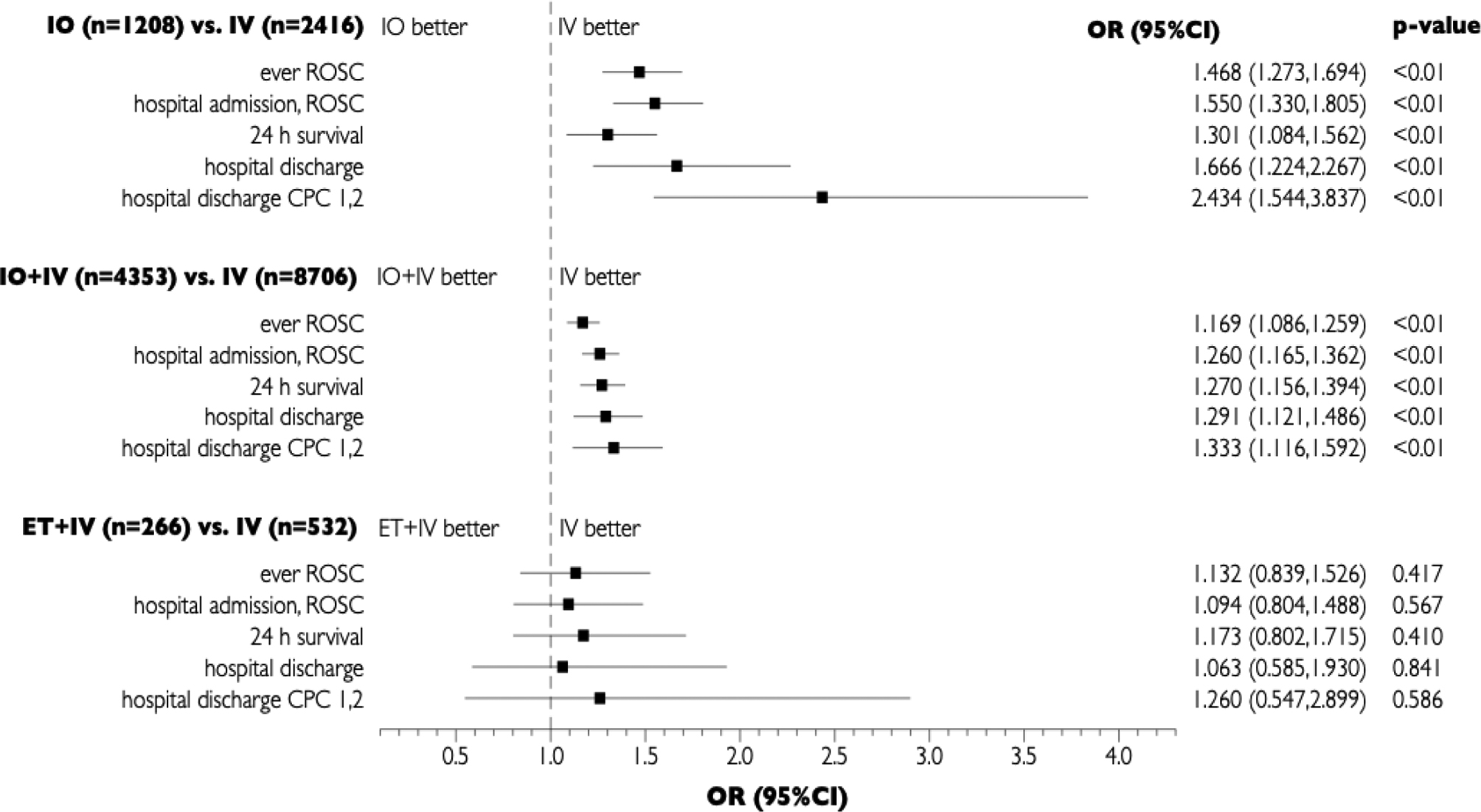
Matched-pair comparisons of clinical outcomes depending on route of administration. IV: intravenous, IO: intraosseous, IO + IV: intraosseous and intravenous, ET + IV: intraosseous and intravenous, OR: odd’s ratio, 95%CI: 95% confidence interval

In pair-matched group comparisons of the primary clinical outcome – hospital discharge with CPC of 1 or 2 – the IV group showed significantly better results compared to IO [odds ratio (OR): 2.43, 95% confidence interval (95% CI): 1.54–3.84, p < 0.01] and compared to IO + IV [OR: 1.33, 95% CI: 1.12–1.59, p < 0.01] (Fig. 2). In contrast, no significant difference was found between IV and ET + IV [OR: 1.26, 95% CI: 0.55–2.90, p = 0.59).

As shown in Fig. 2, equivalent effects were found for all secondary endpoints, too: ROSC at any point, admission to hospital with ROSC, survival at 24 h, survival at 30 days or discharge from hospital. In each comparison, OR significantly favored IV over IO and IV over IO + IV, while no statistically significant difference could be demonstrated for IV vs. ET + IV.

The binary logistic regression model of hospital discharge with good neurological outcome, additionally accounting for age, adrenaline dosage, coronary catheter intervention and provision of mild therapeutic hypothermia, yielded a highly significant effect of vascular access type (χ² = 67.744(3), p < 0.001) with a sufficient amount of explained variance (Nagelkerke’s R² = 0.433). Negative effects could be shown for IO (regression coefficient (r.c.) = − 0.766, p = 0.001) and IO + IV (r.c. = − 0.201, p = 0,028) with no significant effect of ET + IV (r.c. = 0.117, p = 0.770).

## Discussion

The GRR covered over 200,000 cases of OHCA within the 31-year time span from 1989 to 2020. Through pair-matched comparisons of clinical outcome parameters after OHCA, depending on route of drug administration, the present study found clinically relevant and statistically significant differences, generally in favor of the IV access. Analysis of secondary endpoints revealed these effects to be robust for both short term outcomes like admission to hospital with ROSC and long-term outcomes like 30-day survival and discharge from hospital with good neurological outcome.

These findings are seemingly in conflict with existing literature emphasizing the safety and speediness of establishing IO accesses [4, 11, 12]. Some animal models even suggested a pharmacological superiority of IO over IV drug application during CPR [13]. A body of literature on cardiac arrest in swine models reported no effect of access route for adrenaline, comparing IV with humeral and tibial IO [14], and comparing IV with tibial IO [15], nor when comparing vasopressin administration via IV or humeral IO routes [16]. A cardiac arrest study in lambs found no effect in adrenaline administration via tibial IO vs. via central venous access [17].

On the other hand, there are pharmacokinetic studies in animal models of cardiac arrest, suggesting lower plasma levels to be achieved when drugs where applied IO vs. IV [18, 19], confirmed by Hoskins et al. [20], who found an additional decrease in plasma levels in tibial IO vs. sternal IO drug delivery. A 2014 review on IO adrenaline during CPR in animal models therefore recommends proximal over distal IO sites [21].

Retrospective studies in humans demonstrated a time advantage of IO vs. IV access [22, 23], while non-inferiority studies failed to find a significant disadvantage of IO access for clinical outcomes [24]. A current systematic review [25], investigating the effects of venous access type on neurological outcome and survival in OHCA, reported no difference between IV and IO access in the pooled analysis of nine observational studies after correcting for time between cardiac arrest and drug administration. Another systematic review [26], comparing IV and IO routes during cardiac arrest, found limited evidence in favor of IV administration in observational studies and no effect in the subgroup analyses of the randomized controlled trials reviewed.

On the other hand, recent reports from North America [27–33], the UK [34], and France [35], all assessed the IO access under CPR conditions very critically with unfavorable clinical outcome parameters (e.g. ROSC, hospital admission, 30-day survival without neurological deficit). In line with the present GRR data these retrospective studies reported an association of IO treatment during CPR with worse clinical outcomes. Furthermore, a recent systematic meta-analysis [26], also examining the question of application route during CPR, found a probable superiority of IV over IO on the basis of low certainty of evidence. While statistically underpowered for access route analysis, one randomized controlled trial assessing placebo vs. anti-arrhythmic therapy under OHCA [28] found consistently superior clinical outcomes for IV over IO drug administration.

A recent meta-analysis, assessing 23 studies on safety of intravenous peripheral catecholamine administration found a rate of adverse events in under 2% of cases [36].

In summary, while the general safety and rapidness of mere IO placement and the safety of peripheral catecholamine therapy are well documented, the efficacy and effectiveness of IO adrenaline treatment during OHCA remains controversial.

The present findings and literature raising concerns on potential IO inferiority during CPR could have a pharmacokinetic explanation, supported by some of the animal literature referenced above [18–21]. Given the particularly low perfusion pressures present during CPR, transport of adrenaline might prove difficult from the medullary cavity to the place of action within its short half-life of 1 to 2 min, especially for distal IO injection sites like the tibia. Before cardiac and arterial adrenoceptors are reached to elicit the desired arteriolar vasoconstriction as well as inotropic, chronotropic and dromotropic cardiac effects, adrenaline has to exit the medullary cavity, undergo venous return and pass the entire pulmonary circulation. From a pharmacokinetic point of view, an application closer to the target receptors would thus be favorable.

The GRR did not provide information on access site location – specifically, whether an IO access was placed tibially or humerally, or where an IV access was placed. A subgroup analysis of the IO group, challenging the above mentioned hypothesis on proximity to the central circulation was thus not feasible within the present study.

**Fig. 3:**
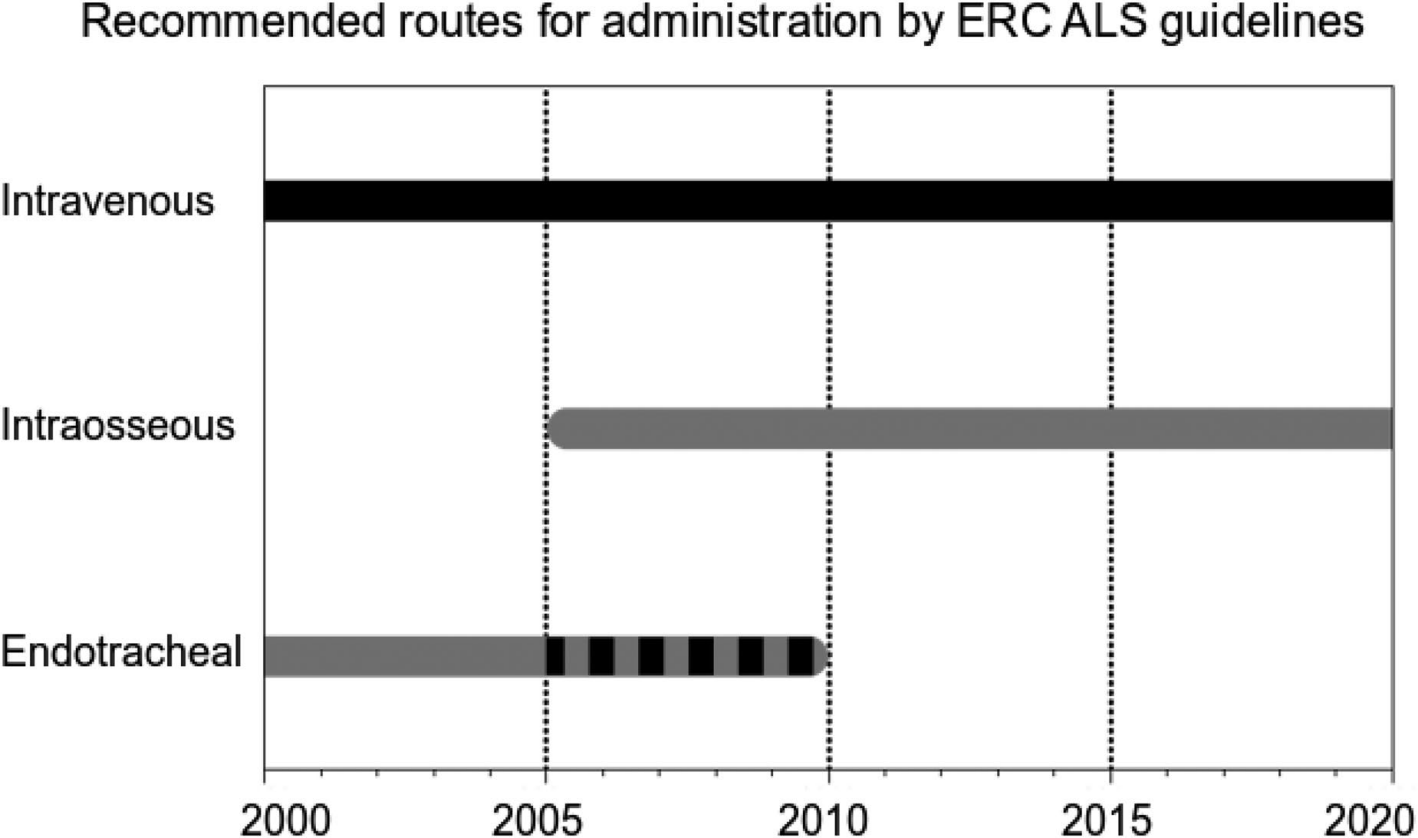
Routes of drug application as recommended by ERC ALS guidelines from 2000 to 2020. Black: first choice, gray: second choice, striped: fallback strategy, ERC: European Resuscitation Council, ALS: advanced life support

One should not forget that the ET administration of adrenaline via an endotracheal tube used to be recommended in international resuscitation guidelines for many years as an alternative to the IV route, providing independence from venous status (Fig. 3). The 2000 ERC guidelines [37] described the ET delivery of adrenaline with higher dosages (2–3 mg ET vs. 1 mg IV) as an equivalent alternative to IV. In 2005, the ERC recommended IO access as the primary alternative to IV, reserving ET administration as an emergency fallback strategy when neither IV nor IO access could be established [38]. Since its 2010 update, ERC guidelines do not recommend the ET route anymore, due to unknown optimal doses and poor predictability of resulting plasma levels [39].

In the event that future studies would confirm a limited efficacy for IO administered emergency medication, in particular for adrenaline during CPR, the risk-benefit-analysis regarding safety, speediness and efficacy of the different routes would have to be re-assessed.

Surprisingly, the present registry analysis suggested an outcome comparability between IV and ET + IV administration of adrenaline. Hypothetically, the decision to discount the ET option could have been made prematurely. If the main reason against recommending the ET route during OHCA CPR was a lack of data on the required dosage, focused research on ET pharmacokinetics during CPR might prove fruitful. Despite not being recommended since 2010, sporadic use of ET adrenaline was detected in the GRR registry as late as 2019.

While safety, speediness and effectiveness of intraosseous access devices are generally not called into question, there might be good reasons to uphold the intravenous access as the gold standard during the specific conditions of OHCA CPR. Nonetheless, whenever the latter is not readily available, a viable and fast alternative access will be pivotal.

In a scenario of ongoing CPR, when the airway has already been successfully secured while IV access has not been established yet, the endotracheal drug administration could potentially present an acceptable alternate route. Before specific recommendations to this effect can be considered, further research on endotracheal dosage requirements is needed.

## Study strength and limitations

As with all registry-based analyses, some limiting factors need to be addressed. First of all, due to the retrospective nature of the study design, control against selection bias through randomization of treatments was not possible. In order to minimize a systematic treatment effect, cases were pair-matched according to the pre-CPR likelihood for ROSC. Internal validation confirmed this approach. Therefore, remaining differences in outcomes cannot be merely explained by postulating a systematic selection bias.

Other potential confounders were implicitly accounted for by referring to the largest available CPR registry in the German-speaking area, hoping to eliminate random effects by collecting a sufficiently large sample. Nonetheless, even this registry did not contain sufficient data to include an exclusively endotracheal treatment group in the analysis, and a substantial number of cases could not be included into the analysis due to incomplete follow-up data. Data on direct comparisons between access routes remain scarce and at times contradictory.

## Conclusions

The GRR data, collected over a 31-year period, provide evidence for using the IV access as primary route during out-of-hospital CPR. IO administration of adrenaline might be less effective. An ET application, while removed in 2010 from international guidelines, could gain in importance as an alternative route again.

## Data Availability

The data sets generated during and/or analyzed during the current study are available from the corresponding author on reasonable request.

## List of abbreviations

ALS: advanced life support
CI: confidence interval
CPC: cerebral performance category
CPR: cardiopulmonary resuscitation
EMS: emergency medical system
ERC: European Resuscitation Council
ET: endotracheal
EuReCa: European Registry of Cardiac Arrest
GRR: German Resuscitation Registry
IO: intraosseous
IV: intravenous
OHCA: out-of-hospital cardiac arrest
OR: odds ratio
RACA: ROSC after cardiac arrest
ROSC: return of spontaneous circulation
